# Diffusion Tensor Imaging Correlates of Concussion Related Cognitive Impairment

**DOI:** 10.3389/fneur.2021.639179

**Published:** 2021-05-24

**Authors:** Angelica C. Gonzalez, Minseon Kim, Zafer Keser, Lamya Ibrahim, Sonia K. Singh, Mohammed J. Ahmad, Omar Hasan, Arash Kamali, Khader M. Hasan, Paul E. Schulz

**Affiliations:** ^1^Department of Neurology, University of Texas McGovern Medical School, Houston, TX, United States; ^2^Department of Diagnostic and Interventional Radiology, University of Texas McGovern Medical School, Houston, TX, United States

**Keywords:** diffusion tensor imaging, concussion, mild traumatic brain injury, cognitive impairment, prognosis

## Abstract

**Introduction:** Cognitive impairment after concussion has been widely reported, but there is no reliable imaging biomarker that predicts the severity of cognitive decline post-concussion. This study tests the hypothesis that patients with a history of concussion and persistent cognitive impairment have fractional anisotropy (FA) and mean diffusivity (MD) values from diffusion tensor imaging (DTI) that are specifically associated with poor performance on the Montreal Cognitive Assessment (MoCA).

**Methods:** Fifty-three subjects (19 females) with concussions and persistent cognitive symptoms had MR imaging and the MoCA. Imaging was analyzed by atlas-based, whole-brain DTI segmentation and FLAIR lesion segmentation. Then, we conducted a random forest-based recursive feature elimination (RFE) with 10-fold cross-validation on the entire dataset, and with partial correlation adjustment for age and lesion load.

**Results:** RFE showed that 11 DTI variables were found to be important predictors of MoCA scores. Partial correlation analyses, corrected for age and lesion load, showed significant correlations between MoCA scores and right fronto-temporal regions: inferior temporal gyrus MD (*r* = −0.62, *p* = 0.00001), middle temporal gyrus MD (*r* = −0.54, *p* = 0.0001), angular gyrus MD (*r* = −0.48, *p* = 0.0008), and inferior frontal gyrus FA (*r* = 0.44, *p* = 0.002).

**Discussion:** This is the first study to demonstrate a correlation between MoCA scores and DTI variables in patients with a history of concussion and persistent cognitive impairment. This kind of research will significantly increase our understanding of why certain persons have persistent cognitive changes after concussion which, in turn, may allow us to predict persistent impairment after concussion and suggest new interventions.

## Introduction

Concussion, which is interchangeably used with “mild traumatic brain injury” (mTBI), is defined as a clinical syndrome of biomechanically induced alteration of brain function, which may involve loss of consciousness (1). As a sequela of concussion, people can develop cognitive impairment, behavioral abnormalities, and mood disorders. The number of TBI-related Emergency Department visits in 2014 was reported as 2.87 million, with 56,800 deaths in the United States (2, 3). Despite its prevalence and severity, no diagnostic or prognostic biomarkers unique to concussion have been validated, which has greatly hindered our ability to test early interventions (4).

Previous studies have revealed that diffuse axonal injury (DAI) is a critical pathologic finding in concussion that cannot be detected by CT or conventional MRI (5–8). Diffusion tensor imaging (DTI) has been widely used in the study of concussion because it can reliably detect the microstructural white matter changes found in DAI. The two most commonly used DTI parameters are fractional anisotropy (FA) and mean diffusivity (MD) (9, 10). FA quantifies the directionality of water diffusion, which ranges from 0 (isotropic) to 1 (anisotropic). MD measures the total diffusion rate in all directions within a voxel. White matter damage, as seen in DAI, results in fewer microstructural elements that limit diffusion, thereby decreasing the FA and increasing the MD. In addition, the Montreal Cognitive Assessment (MoCA) has proven to be a promising tool due to its ability to screen for occult memory impairment in patients with post-concussive syndrome and mTBI with high sensitivity.

The purpose of this study was to test the hypothesis that patients with cognitive impairment post-concussion have DTI-derived neuroimaging biomarkers that are specifically associated with poorer MoCA scores.

## Methods

Fifty-three subjects (19 females) with a history of concussion were evaluated at UTHealth Neurosciences Neurocognitive Disorders Center in Houston, Texas. The concussion was secondary to various etiologies, including sports-related, car accidents, and falls, that lead to varying degrees of persistent cognitive impairment and neuropsychologic symptoms were included in this study (Tables 1, 2 for demographics, characteristics, and symptoms). The subjects had a MoCA and MR imaging, including T1w, fluid-attenuated inversion recovery (FLAIR), and diffusion-weighted imaging (DWI) sequences.

**Table 1:** Demographic characteristics and description of subjects with post-concussive symptoms.

**Characteristics (*n* = 53)**	**Frequency (%)**
Age, Median [IQR^*^]	55 [36–68]
**Sex**	
Female	19 (35%)
Male	34 (64%)
**Number of Trauma**	
Single	20 (38%)
Multiple	33 (62%)
**Mechanism of Trauma**^**a**^	
Sports-related	27 (51%)
Falls	9 (17%)
Motor vehicle accident	14 (26%)
Hit head against surface	7 (13%)
Physical abuse	1 (2%)
Suicidal attempt	1 (2%)
Loss of Consciousness	25 (47%)
MoCA Score, Median [IQR^*^]	26 [20–27]
**Cognitive Risk Factors other than TBI**	
Family history of dementia	15 (28%)
Cardiovascular^b^	27 (51%)
Depression/anxiety	23 (43%)

* *IQR: inter-quartile range*.

a *Some patients had multiple machanisms of trauma and multiple cognitive risk factors*.

b *Cardiovascular risk factors include tobacco use, BMI > 30, hypertension, hyperlipidemia, and diabetes mellitus*.

**Table 2:** Description of symptoms.

Complaints^*^ (*n* = 53)	**Frequency (%)**
**Behavioral**	
Sleep difficulties	20 (38%)
Personality changes	24 (45%)
**Cognitive**	
Memory impairment	53 (100%)
Inattention	13 (24%)
Word finding difficulty	14 (26%)
**Somatic**	
Headache	17 (32%)
Vertigo	5 (9%)
**Emotional**	
Depression	3 (6%)
Anxiety	7 (13%)

* *Some patients had multiple complaints*.

### Image Acquisitions and Analyses

Whole-brain MRI data were acquired on a Philips 3.0 T Intera scanner using a SENSE receive head coil. Both T1-weighted and FLAIR sequences had a spatial resolution of 1 mm × 1 mm × 1 mm, and field-of-view was 256 × 256 mm. Diffusion-weighted image (DWI) data were acquired axially using a single-shot multi-slice 2-D spin-echo diffusion sensitized and fat-suppressed echo-planar imaging (EPI) sequence, with the balanced Icosa21 tensor encoding scheme (11). The b-factor was 1,000 s mm−2, TR/TE 7,100/65 ms, FOV 256 × 256 mm, and slice thickness/gap/#slices = 3 mm/0 mm/44. The EPI phase encoding used a SENSE k-space undersampling factor of two, with an effective k-space matrix of 128 × 128, and an image matrix after zero-filling of 256 × 256. The constructed image spatial resolution for the DWI data was = 1 × 1 × 3 mm.

We performed whole-brain atlas-based DTI segmentation through MRICloud software (168 regions) and obtained FA and MD values (https://braingps.mricloud.org/) (12). We performed lesion segmentation on FLAIR sequences through volBrain software (https://www.volbrain.upv.es/) (13). Lesion load was then converted to the percentage of total intracranial volume (ICV) [formula = lesion volume (ml)/intracranial volume (ICV) × 100]. Both DTI and lesion segmentations were inspected on a case-by-case basis for anatomical accuracy.

### Statistical Analyses

Histogram plots were utilized to identify distribution patterns. Descriptive statistics were used to compute the means and standard deviations (SD) or the medians and the range between first and third quantiles if not normally distributed. We then conducted a random forest-based recursive feature elimination (RFE) with 10-fold cross-validation in the entire dataset for all the regions FA and MD values to compute the importance for each predictor and remove redundant predictors of MoCA scores. The central premise using a feature selection technique is that data contains some features that are either redundant or irrelevant; thus, they can be removed without incurring much loss of information (14). In practice, the backward elimination regression model method that calculates the importance of each independent variable and removes the ones with the least importance based on root mean square error (RMSE) metric. As it is not a machine-learning algorithm, training or test dataset was not used. After the removal of the redundant independent variables, the partial correlation adjusted for age and lesion load was performed to identify the correlations between MoCA scores and remaining DTI scores. We corrected our analysis for white lesion load as leukoaraiosis is independently associated with cognitive decline (15). After obtaining *p*-values, the false discovery rate (FDR) analysis of 5% was also conducted for multiple comparison analyses and only corrected *p*-values were reported. R statistical package was used for the statistical analyses.

## Results

### Study Design and Participants

Fifty-three subjects who suffered from a concussion and had persistent symptoms were included in this study (34 males and 19 females). Twenty-seven patients had a history of concussion related to sports, with the majority being football players. Other mechanisms of concussion included: motor vehicle accident (14), falls (9), hitting the head against a surface (7), suicide attempts (1), and physical abuse (1). Thirty-three patients reported multiple concussions, and 25 reported a loss of consciousness from their trauma. The last concussion before they presented to the clinic varied from 1 month to 45 years. Most patients experienced symptoms months or years before they consulted. A summary of the patient's demographics and description can be found in Table 1, and a more detail information can be found in Supplementary Material.

Forty-eight patients were experiencing memory problems as their main reason for consult, and it was associated with symptoms such as headache, changes in their sleep, personality and or behavioral changes, difficulty finding words, new onset of mood disorder, and vertigo. Five other patients reported headache (2), inattention (2), and vertigo (1) as their chief complaints, with memory problems as an associated symptom. Table 2 shows patient's self-reported symptoms. Forty-two patients had other cognitive risk factors, such as obesity, hypertension, hyperlipidemia, hypertriglyceridemia, diabetes, cardiovascular disease, depression, anxiety, family history of dementia or tobacco use (Table 1).

Age, MoCA scores, and lesion load were not normally distributed, whereas DTI values were normally distributed per histogram plots. Median age was 55 (1st quantile = 36- 3rd quantile = 68), median MoCA was 26 (20–27), and median lesion load was 0.06 (0.02–0.25).

**Figure 1:**
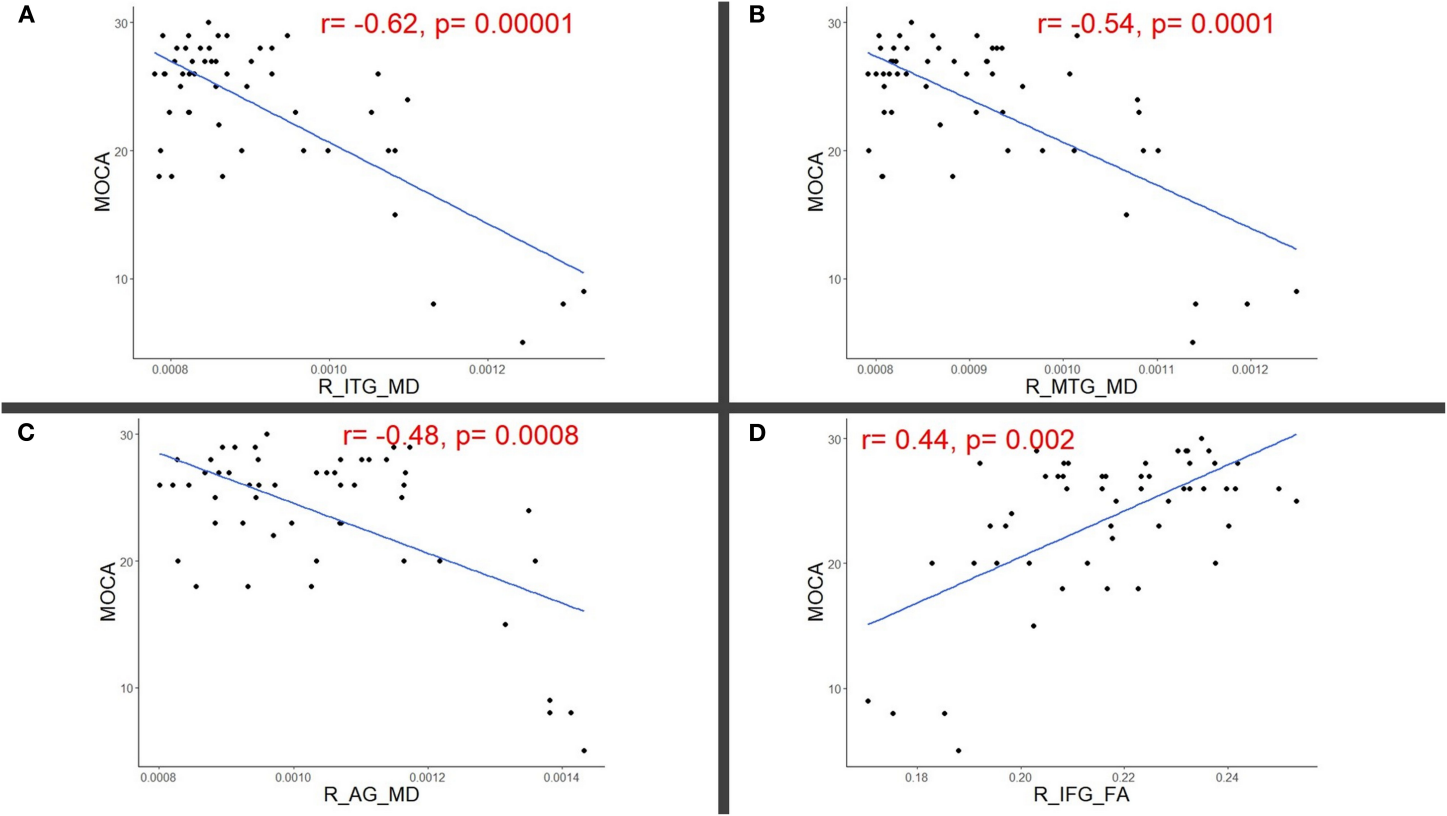
Scatter plots illustrating significant partial correlations (adjusted for age and lesion load) between Montreal cognitive assessment (MoCA) and right (R) **(A)** Inferior temporal (ITG), **(B)** Middle temporal (MTG), **(C)** Angular (AG), and **(D)** Inferior frontal gyri (IFG).

RFE showed that 12 DTI variables were found to be important predictors of MoCA scores and were included in the correlation analyses; the right inferior temporal gyrus (ITG) MD (0.90 ± 0.13 × 10-3 mm^*^mm/s), the right middle temporal gyrus (MTG) MD (0.91 ± 0.11 × 10-3) and FA (0.20 ± 0.01), the right angular gyrus (AG) MD (1.05 ± 0.17 × 10-3 mm^*^mm/s), the right inferior frontal gyrus (IFG) FA (0.22 ± 0.02), the right entorhinal cortex MD (0.90 ± 0.13 × 10-3), the left fornix FA (0.39 ± 0.09), the left nucleus accumbens MD (1.13 ± 0.22 × 10-3), right splenium of the corpus callosum FA (0.61 ± 0.04), and bilateral tapetum of corpus callosum FAs (right = 0.45 ±0.08, left = 0.52 ± 0.09). For these values, the partial correlation analyses corrected for age and lesion showed a significant correlation between MoCA scores and right fronto-temporal regions; right ITG MD (*r* = −0.62, *p* = 0.00001), right MTG MD (*r* = −0.54, *p* = 0.0001), right AG MD (*r* = −0.48, *p* = 0.0008), right IFG FA (*r* = 0.44, *p* = 0.002) whereas the remaining values did not show significant correlations with MoCA after FDR corrections (*p* > 0.05). Significant correlations were highlighted in Figure 1.

## Discussion

To our knowledge, this is the first study to investigate the correlation between MoCA scores and DTI variables using atlas-based methods in patients with a history of concussion and persistent cognitive impairment. The MoCA is a quick, convenient, and sensitive screening tool for cognitive impairment. Its administration consists of 12 individual tasks grouped into seven cognitive domains: (1) visuospatial/executive; (2) naming; (3) attention; (4) language; (5) abstraction; (6) memory and (7) orientation. Memory, attention, and visuospatial functions are the most frequently affected domains in TBI (3). We analyzed 53 patients' data from medical records in an outpatient setting. The average time between the evaluation of the patients and the last concussion was approximately 2.8 years. Hence, our analysis reflects the relationship between chronic brain changes after TBI and the subject's performance on the MOCA.

Concussion or mTBI is a clinical diagnosis due to the absence of validated diagnostic biomarkers (4). Predicting cognitive outcomes is vital for early rehabilitation, medical management, and experimental therapies designed to improve long-term prognosis. There is a dearth of standardized techniques for the detection and prediction of cognitive outcomes after mTBI.

It is important to note that several studies have applied other methods to have a better understanding of the anatomical changes post TBI and how these alterations can affect cognitive performance (16). It has been well established that a reduction of total brain volume and cerebral atrophy are common sequelae of TBI (17–20). Prior publications have assessed these subtle volumetric changes to predict a clinical outcome post TBI. Most of the morphometric measures that have been published are based on the segmentation techniques available in FreeSurfer (http://surfer.nmr.mgh.harvard.edu/). Warner et al. assessed the relationship between the cognitive outcomes in 24 patients post traumatic axonal injury (TAI) with white matter integrity and regional brain volumes (21). Their work concluded that regional brain volumes were correlated with deficits in neuropsychological outcomes and that volumes of some gray matter structures were more strongly associated with damage to related white matter tracts in the chronic phase than in the acute phase. Numerous other studies have identified that certain brain regions such as the thalamus and hippocampus are selectively vulnerable to atrophy after trauma and have significant value when predicting functional outcome (21, 22). Therefore, brain volume and cortical track integrity are useful tools when assessing cognitive prognosis in post TBI patients. In this study, we focused on microstructural changes and its correlation with MOCA scores.

Numerous studies have investigated the role of DTI in mTBI. In 2002, Arfanakis et al. (6) described five patients with mTBI who underwent DTI in the first 24 h of presentation to the Emergency Department and identified regions of diffuse axonal injury that appear normal with conventional neuroimaging. However, DTI in the acute phase can show changes due to vasogenic edema that can be reversible and therefore does not reflect the chronic brain changes related to cognitive impairment. In the last decade, over a 100 publications have demonstrated the value of DTI at detecting microstructural disruption in concussion (23). However, this study represents an advance over previous studies because it investigated patients who are in the chronic phase post-concussion, which eliminates possible misleading findings in the acute and subacute phases. Also, our DTI analysis reflects white as well as gray matter changes, as opposed to most previous studies that have analyzed only white matter changes. Finally, an important advance here is that the MoCA was performed around the same time as the brain DTI so that the correlations between them are more reliable.

Our findings demonstrated a negative correlation between MoCA scores and MD values in the right inferior temporal gyri, middle temporal gyri, and angular gyri. We found a positive correlation between MoCA scores and FA values in the right inferior frontal gyri. Therefore, areas exhibiting loss of integrity reflected by abnormal FA and MD values, which significantly correlated with MOCA scores, were mostly in the right frontotemporal area. Notably, this result also indicates that the changes in MOCA scores after concussion are not due to impaired language because the right hemisphere typically does not affect language function, outside of left handers.

The temporal lobe of the brain has several brain structures that are critical for cognitive functions. It subdivides into the superior, middle, and inferior temporal gyrus (STG, MTG, ITG). Between these subdivisions and between different parcellations of the frontal, parietal and occipital lobes, there are functional white matter connections (structural connectivity) that are essential for memory and visuospatial performance (24). The ITG contains the temporal area 2 anterior (TE2a) and the temporal area 2 posterior (TE2p) that appear to function in vision. The MTG contains the perirhinal cortex, which contributes to declarative memories and semantic knowledge (25). Declarative memories are those that can be consciously thought of and verbalized. Some studies speculate that the medial temporal lobe is crucial for semantic memory—the ability to recall general facts about the world (26). On the other hand, the role of the right inferior frontal gyrus (IFG) has been strongly associated with switching attention from one object to another by inhibiting the previously attended locus (27–29). As above, memory, attention, and visuospatial functions are the most frequently affected domains in TBI (3), and here we identified a clear correlation between those areas of the brain that exhibited significant DTI findings and the most commonly affected cognitive domains in TBI.

Our results suggest that lower MoCA scores are associated with higher MD in the right temporal regions and decreased FA in the right frontal region. Our study supports previous findings that have established an association between post-concussion syndrome and increased MD and decreased FA in DTI. However, these findings have been inconsistent when trying to identify the areas of the brain that are affected, and our results differ from some previous studies. For example, there have been suggestions of increased MD in the corpus callosum (30–32); the left uncinate fasciculus (33); the inferior fronto-occipital fasciculus, the inferior longitudinal fasciculus, the superior longitudinal fasciculus, the corticospinal tract and the left anterior thalamic radiation (31).

Decreased FA in different brain areas has also been inconsistent. Studies have suggested decreased FA in the whole brain (34); the corpus callosum (32, 33, 35, 36); the right anterior corona radiata, internal capsule (anterior limb), fornix, and medial superior frontal gyrus (35); the pontine tegmentum (37); and the left uncinate fasciculus and bilateral superior thalamic radiations (33). These discrepant results could be attributed to variations in the time interval between injury and imaging and differences in study design and analytic techniques. Consequently, no standard DTI biomarker is identified for PCS diagnosis and prediction (38).

Our findings extend previous ones and present strong evidence for right frontotemporal changes underlying persistent cognitive symptoms after concussion because (1) there is a clinico-pathologic correlation between our cognitive and imaging data, (2) the affected regions identified in DTI match the symptoms reported by the patients and (3) the cognitive weakness domains on testing correspond to the function of the affected brain areas exhibiting DTI changes. We would suggest, then, that increased MD and decreased FA in the right frontotemporal regions may predict low MoCA scores in people who have suffered from mTBI. These results may be helpful when assessing patients complaining of cognitive impairment who have a history of concussion.

There are some limitations to this study. For example, diffusion tensor metrics are sensitive, but non-specific markers for microstructural changes of the brain parenchyma, which can be altered in many brain pathologies including infection, inflammation or trauma. Our study included a heterogeneous population, and subjects had other cognitive risk factors such as hypertension, hyperlipidemia and diabetes. These comorbidities are also known to cause white matter changes (39). Therefore, we cannot rule out a contribution by those risks factors to white matter damage and poor MoCA performance. Although we have not controlled our analysis for all the risk factors for cognitive impairment, we qualitatively presented them in a detailed manner in Table 1. On the other hand, many patients were young, and lacked these risk factors, and still had the changes we noted on DTI imaging. Other limitations of our study include small sample size, differences in the interval from injury to imaging, and lack of a control group. The small sample size meant that our study lacked the power to perform in-depth analyses for MOCA subscores and their DTI correlates; hence, we only investigated global cognitive impairment and it's DTI correlates. A larger, future study would allow for important subscore analyses.

Going forward, it will be important to determine which components of concussion are potentially reversible, and which may be irreversible. Moreover, additional, large-scale, longitudinal studies and translational research are needed to further explore DTI as a reliable prognostic indicator. Functional imaging, such as PET scans, SPECT scans, and evoked potentials, have shown inconsistent results across studies, while a limited number of studies have found promise in the application of MR spectroscopy in detecting diffuse axonal injury and post-concussion syndrome (40, 41). Further research into potential biochemical markers, such as neurofilaments, neuron specific enolase, S100B, and ferritin (39), which are correlated with imaging and cognitive assessment results, would also broaden our insight into diagnostic, prognostic, and therapeutic options for mTBI.

## Data Availability Statement

The original contributions presented in the study are included in the article/Supplementary Material, further inquiries can be directed to the corresponding author/s.

## Ethics Statement

The studies involving human participants were reviewed and approved by Committee for the Protection of Human Subjects. Written informed consent from the participants' legal guardian/next of kin was not required to participate in this study in accordance with the national legislation and the institutional requirements.

## Author Contributions

All authors listed have made a substantial, direct and intellectual contribution to the work, and approved it for publication.

## Conflict of Interest

The authors declare that the research was conducted in the absence of any commercial or financial relationships that could be construed as a potential conflict of interest.
