# Supplementary material for: Diffusion Tensor Imaging Correlates of Concussion Related Cognitive Impairment
Source: Front Neurol. 2021 May 24;12:639179. doi: 10.3389/fneur.2021.639179 (PMC8180854; doi:10.3389/fneur.2021.639179)
Supplement: Supplementary file 1 [file Table_1.DOCX]

Supplementary Material

**Table 1: Demographic characteristics and description of female subjects with post-concussive symptoms.**

| **Age range (in years)** | **Chief complaint** | **Symptoms self – report** | **Mechanism of trauma** | **Single vs. Multiple Trauma** | **LOC after injury** | **Last concussion Before presentation** | **Cognitive Risk factors other than TBI** | **MoCA score** |
| --- | --- | --- | --- | --- | --- | --- | --- | --- |
| 16-20 | Attention deficit | Sleep changes, anxiety | Hit head against surface | Single | No | 2 years | None | 30 |
| 16-20 | Memory impairment | HA, Anxiety, dizziness | MVA, sport related (Soccer) | Multiple | No | 11 months | None | 29 |
| 16-20 | Memory impairment | HA, Attention problems | Sports related (Gymnastic) | Multiple | Yes | 4 years | AD in family | 29 |
| 21-25 | Poor concentration | HA | MVA, sport related | Multiple | Yes | 4 months | Anxiety, depression | 26 |
| 36-40 | Memory impairment | Word finding difficulty, attention deficit | Suicidal attempt | Single | No | 20 years | Depression | 29 |
| 46-50 | Memory impairment | Word finding difficulty, sleep changes, attention deficit, depression | MVA | Single | No | Unknown | BMI >30, HTN | 21 |
| 46-50 | Memory impairment | Word finding difficulty, attention deficit | Hit head against surface | Multiple | Yes | 3 years | AD in family, depression, HLD, HTN | 28 |
| 46-50 | Memory impairment | HA, sleep changes, word finding difficulty | MVA | Single | No | 5 months | Depression, CVA, HLD | 25 |
| 51-55 | Memory impairment | Personality changes | Fall | Single | Yes | 7 years | None | 27 |
| 56-60 | Memory impairment | HA, anxiety, sleep changes, personality changes, hallucinations | Fall, physical abuse | Multiples | Yes | 4 years | AD in family, tobacco use, BMI >30 | 8 |
| 56-60 | Memory impairment | HA | Fall | Single | Yes | 10 months | Alcohol and drug use, HLD | 26 |
| 56-60 | Memory impairment | HA | MVA | Single | Yes | 16 years | None | 25 |
| 56-60 | Memory impairment | Sleep changes | Fall | Single | No | 1 year | HLD | 29 |
| 61-65 | Memory impairment | HA | Fall | Single | No | 5 months | AD in family | 27 |
| 61-65 | Memory impairment | Word finding diff., personality changes | Sports related (Biking) | Single | Yes | 1 year | AD in family, HLD | 28 |
| 61-65 | Memory impairment | Word finding difficulty, attention deficit, personality changes, vertigo | MVA | Single | Yes | 15 months | Depression and anxiety | 25 |
| 66-70 | Memory impairment | Word finding difficulty | MVA | Single | Yes | 28 years | HLD | 25 |
| 66-70 | Memory impairment | Attention deficit, Depression, anxiety | MVA | Single | Yes | 4 years | HLD, AD in family | 18 |
| 66-70 | Memory impairment | None | Fall | Single | No | Unknown | HLD, depression, DM, BMI >30, HTN, AD in Family | 23 |

**Table 2: Demographic characteristics and description of male subjects with post-concussive symptoms.**

| **Age range (in years)** | **Chief complain** | **Symptoms self – report** | **Mechanism of trauma** | **Single vs. Multiple Trauma** | **LOC after injury** | **Last concussion Before presentation** | **Cognitive Risk factors other than TBI** | **MoCA score** |
| --- | --- | --- | --- | --- | --- | --- | --- | --- |
| 16-20 | Memory impairment | Attention deficit, sleep changes, irritability, vertigo, depression | Sports related | Multiple | No | 3 months | None | 26 |
| 16-20 | Memory impairment | Hallucinations, personality changes | Sports related (football player) | Multiple | No | 1 year | Depression, anxiety | 18 |
| 16-20 | Memory impairment | HA, sleep changes, personality changes, vertigo | Sports related (football player) | Multiple | Yes | 3 months | None | 28 |
| 16-20 | Memory impairment | HA, Sleep changes, attention deficit, personality changes | Sports related (football player) | Multiple | No | 3 years | Depression, anxiety | 23 |
| 16-20 | HA | Memory impairment, sleep changes | Sports related (football player) | Multiple | No | 1 month | HTN, BMI >30 | 28 |
| 16-20 | Memory impairment | HA, sleep changes | Sports related (football player) | Multiple | Yes | 1 month | HTN | 21 |
| 25-30 | Memory impairment | HA, sleep changes, personality changes | Sports related (football player) | Multiple | No | 3 months ago | None | 25 |
| 25-30 | Memory impairment | Attention deficit | Sports related (football player) | Multiple | No | Unknown | Depression. anxiety, AD in family | 28 |
| 31-35 | Memory impairment | None | Sports related (football player) | Multiple | Yes | 10 years | Depression. anxiety, | 26 |
| 31-35 | Memory impairment | Personality changes, sleep changes, depression, anxiety | Sports related (bull rider) | Multiple | Yes | Unknown | None | 27 |
| 36-40 | Memory impairment | HA, vertigo, Depression | Sports related (football player) | Multiple | Yes | 4 years | Anxiety, HLD, HTN | 23 |
| 41-45 | Memory impairment | Anxiety | MVA, Hit head against surface | Multiple | No | 2 months | Depression | 20 |
| 41-45 | Memory impairment | Anxiety | Sports related (Football player) | Multiple | No | 20 years | Depression, HLD, BMI >30 | 26 |
| 41-45 | Memory impairment | Sleep problems, word finding difficulty, personality changes | Hit head against surface | Multiple | Yes | 12 years | BMI >30, anxiety and depression, PTSD | 27 |
| 51-55 | Memory impairment | Depression, anxiety, personality changes | Sports related (Football player) | Multiple | No | Unknown | AD in family | 27 |
| 51-55 | Memory impairment | Sleep changes, personality changes | Fall and sport related | Multiple | Yes | 2 years | BMI >30, HTN, Hx of depression | 18 |
| 51-55 | Memory impairment | None | Fall | Multiple | No | Unknown | Depression | 29 |
| 51-55 | HA | Memory impairment, sleep changes, personality changes | MVA | Single | Yes | 1 year | BMI >30, anxiety | 27 |
| 51-55 | Memory impairment | Depression, HA, Sleep changes, personality changes | Sports related (Football) | Multiple | Yes | 2 years | BMI >30, HTN, anxiety | 24 |
| 56-60 | Memory impairment | Sleep changes, attention deficit, word finding difficulty. | Sports related (Football) | Multiple | No | 3 years | Depression. anxiety, AD in family | 27 |
| 56-60 | Memory impairment | Word finding difficulty, sleep changes, personality changes, depression | Sports related (Biking) | Single | No | 3 months | None | 26 |
| 56-60 | Memory impairment | HA, Confusion | Fall | Single | No | 10 years | BMI >30, HTN, Tobacco use, NPH | 20 |
| 61-65 | Vertigo | Memory problems, personality changes, depression | Hit head against surface | Multiple | Yes | Unknown | HTN, DM, BMI>30, HLD | 20 |
| 66-70 | Memory impairment | Word finding difficulty, personality changes | MVA, sports related (Football) | Multiple | No | Unknown | DM, Dementia, BMI >30, HTN, HLD, AD in family | 26 |
| 66-70 | Memory impairment | Personality changes | Hit head against surface | Single | Yes | 5 years | None | 22 |
| 66-70 | Memory impairment | Word finding difficulty, personality changes | Sports related (football player and ski) | Multiples | Yes | 4 months | HTN, DM, HLD, HTN, AD in family Hx. | 24 |
| 66-70 | Memory impairment | Sleep changes, HA, personality changes | MVA | Single | No | 2 weeks | HTN, DM, BMI >30 | 27 |
| 66-70 | Memory impairment | Sleep changes | Sports related (football player) | Multiple | No | 45 years | Depression | 5 |
| 71-75 | Memory impairment | Word finding difficulty, personality changes, hallucinations | MVA, and sport related (Box) | Multiple | No | 4 years | Depression. anxiety,, AD in family | 8 |
| 71-75 | Memory impairment | None | Hit head against surface, falls | Multiple | Yes | 10 months | DM, alcohol abuse, HLD, AD in family | 20 |
| 71-75 | Memory impairment | None | Sports related (football player) | Multiple | Yes | Unknown | HLD, AD in Family | 20 |
| 76-80 | Memory impairment | Word finding difficulty | Sports-related (football player) | Multiple | No | Unknown | Depression, BMI >30, HTN, DM, HLD | 15 |
| 76-80 | Memory impairment | None | Sports-related (football player) | Multiple | No | Unknown | HLD, depression. anxiety, | 9 |

*MVA: motor vehicle accident, HA: headache, AD: Alzheimer disease, BMI: body mass index, DM: diabetes mellitus, HTN: hypertension, HLD: hyperlipidemia, CVA: cardiovascular accident.
